# Transfer of Anti-Rotavirus Antibodies during Pregnancy and in Milk Following Maternal Vaccination with a Herpes Simplex Virus Type-1 Amplicon Vector

**DOI:** 10.3390/ijms18020431

**Published:** 2017-02-16

**Authors:** Anita F. Meier, Mark Suter, Elisabeth M. Schraner, Bruno M. Humbel, Kurt Tobler, Mathias Ackermann, Andrea S. Laimbacher

**Affiliations:** 1 Institute of Virology, Vetsuisse Faculty, University of Zurich, 8057 Zurich, Switzerland; anita.meier@uzh.ch (A.F.M.); kurt.tobler@uzh.ch (K.T.); mathias.ackermann@uzh.ch (M.A.); 2 Immunology Division, Vetsuisse Faculty, University of Zurich, 8057 Zurich, Switzerland; m.suter@vetadm.uzh.ch; 3 Institutes of Veterinary Anatomy and Virology, University of Zurich, 8057 Zurich, Switzerland; elisabeth.schraner@uzh.ch; 4 Electron Microscopy Facility, University of Lausanne, 1015 Lausanne, Switzerland; bruno.humbel@unil.ch

**Keywords:** HSV-1 amplicon vector, rotavirus-like particles, lactogenic antibody transfer

## Abstract

Rotaviruses (RVs) are important enteric pathogens of newborn humans and animals, causing diarrhea and in rare cases death, especially in very young individuals. Rotavirus vaccines presently used are modified live vaccines that lack complete biological safety. Previous work from our laboratory suggested that vaccines based on in situ produced, non-infectious rotavirus-like particles (RVLPs) are efficient while being entirely safe. However, using either vaccine, active mucosal immunization cannot induce protective immunity in newborns due to their immature immune system. We therefore hypothesized that offspring from vaccinated dams are passively immunized either by transfer of maternal antibodies during pregnancy or by taking up antibodies from milk. Using a codon optimized polycistronic gene expression cassette packaged into herpesvirus particles, the simultaneous expression of the RV capsid genes led to the intracellular formation of RVLPs in various cell lines. Vaccinated dams developed a strong RV specific IgG antibody response determined in sera and milk of both mother and pups. Moreover, sera of naïve pups nursed by vaccinated dams also had RV specific antibodies suggesting a lactogenic transfer of antibodies. Although full protection of pups was not achieved in this mouse model, our observations are important for the development of improved vaccines against RV in humans as well as in various animal species.

## 1. Introduction

Rotavirus (RV) is an enteric pathogen of humans and other vertebrates. Symptoms of RV infection include gastroenteritis, leading to dehydrating diarrhea and death in some cases (for reviews see [1,2]). More than an estimated 700,000 children under the age of five died of diarrhea in 2011. Importantly, 28% of these fatal diarrhea cases were associated with RV [3].

Although there are licensed RV vaccines available, their protective effect against severe RV induced diarrhea was found to be extremely region specific with a good efficiency in developed regions (90.6%). By contrast, in other regions, such as in sub-Saharan Africa, protection was only 46.1% [4]. Globally approved vaccines include either live attenuated (Rotarix, RV1; GlaxoSmithKline Inc., Rixensart, Belgium) or reassortant RV strains (RotaTeq, RV5; Merck and Co., Inc., Whitehouse Station, NJ, USA). Hence, both vaccines are based on replicating viral strains and are thus not safe by nature. A case report from 2010 described the RV transmission from a RotaTeq vaccinated child to its unvaccinated sibling. This vaccine associated infection resulted in gastroenteritis that required emergency care. The transmitted RV strain was a reassorted vaccine-derived strain. Reassortment may have resulted in the recovery of virulence [5]. In addition, both RV vaccines were found to be associated with a slightly increased risk of intussusception after the primary vaccination [6]. Therefore, it is necessary to develop more efficient and in particular safer vaccines. Here, we present our findings on an alternative vaccine approach that ultimately aims to protect children from RV infection-associated symptoms in their early time of life.

Virus-like-particles (VLPs) are composed of one or several viral capsid proteins that self-assemble into a spatial conformation that resembles the structure of the original virus. Virus-like-particles comprise a high density of epitopes on their surface, which can be recognized by antigen presenting cells (APCs) and thereby stimulate a humoral and cellular immune response through similar pathways as the complete, pathogenic virus [7]. Virus-like-particles do not contain any genetic material and thus do not replicate and are therefore considered safe. Vaccines based on purified VLPs, for example Gardasil (Merck and Co., Inc., Whitehouse Station, NJ, USA) and Cervarix (GlaxoSmithKline, Inc., Brentford, UK) for human papilloma virus (HPV) immunization and Recombivax HB against hepatitis B virus (HBV; Merck and Co., Inc., Whitehouse Station, NJ, USA), are on the market [8].

The mature infectious RV particles are non-enveloped, triple-layered capsids composed of the structural proteins VP2, forming the inner most shell, VP6 assembling as the middle layer and VP7 as third and most outer layer that forms a net-like structure adhering to VP6 [1]. For the attachment to the host cell, the capsids also contain VP4 spikes that protrude through VP7 pores and attach to the VP6 layer. To produce VLPs of RV, many expression vectors exist and several vaccine candidates have been proven to be effective against RV induced diarrhea or virus shedding in small animal models. Triple-layered VLPs containing VP2, VP6 and VP7 [9,10] or double-layered VLPs consisting of VP2 and VP6 are examples of this approach [11]. Moreover, subunit vaccines produced of VP6 in eukaryotic cells [12,13] or VP2 and VP6 expressed as soluble proteins in *Escherichia coli* and assembled into VLPs [14] showed protection against RV induced disease. However, the assembly of RV structural proteins into rotavirus like particles (RVLPs) requires the simultaneous expression of several recombinant RV genes in a single cell followed by multiple purification steps of these RVLPs. These are challenging steps in the production of a stable, mass-produced vaccine.

To circumvent these problems but maintaining the ease of application and safety, we decided to trigger the immune response using RVLPs produced within cells of the vaccinee but in the absence of RV genome synthesis. We used the herpes simplex virus type-1 (HSV-1) amplicon vector to deliver a DNA cassette with a single polycistronic messenger RNA which contains the coding sequences of the three capsid proteins VP2, VP6, and VP7, separated by internal ribosome entry sites for the production of the RVLPs. The HSV-1 amplicon vector system with its high transgenic capacity of up to 150 kb is safe as the transgenes are unable to replicate [15], but allow delivery of synthetic DNA encoding any antigen of interest [16]. With this system, the time consuming and complex purification of VLPs is not required. Herpes simplex virus type-1 amplicon vector transduction triggering RVLP production was shown in previous studies from our laboratory but VP2 and VP7 were dramatically underrepresented compared to VP6 [17].

Structural vaccinology approaches involve the engineering of immunogens using a combination of structural biology and immunology with the idea that the protective antigens are optimized and simplified for inclusion in vaccine formulations to enhance efficacy, stability and delivery to obtain a stronger immunogenicity and hence a broader protection. An important goal of structural vaccinology is the conformational stabilization of an antigen based on its native three-dimensional structure to induce an efficacious immune response. Based on these principles, we aimed to increase the amounts of VLP-generating and immunizing proteins synthesized in situ, we chose to alter the sequence of viral genes in the amplicon vector and optimized the amino acid sequence to the codon usage of human cells.

Protection of newborn human and mice against various pathogens is supported by placental transfer of antibodies from mother to child and, additionally, after birth via the lactogenic route [18,19,20]. Notably, the number of placental layers between mothers and their fetuses is different for various mammals, which affects or inhibits the ability to transfer antibodies during pregnancy. The mammals cluster into three groups, with humans belonging to group 1, where, due to their single layer placenta type, the major amount of immunoglobulin is transferred to the fetus prior to parturition [21]. In group 2, with mice and dogs as examples, the immunoglobulins are transferred both through the placental and the lactogenic pathway. The efficiency by which different isotypes of antibodies are transferred to the milk differs between the individual species. Although being aware of these differences, it was of interest to observe the general potency of our vaccine type in a mouse model. We thus studied the kinetics of anti-RV antibody production in the sera of mice as well as in their milk. Moreover, we tested for prepartum antibody transfer compared to the postpartum transfer by milk. Finally, we also tested the protective effect of passive antibody transfer in the context of a challenge infection.

Here, we show that the transduction of cells in vitro with the HSV-1 amplicon vector that delivers a DNA cassette encoding a single polycistronic messenger RNA, which contains the coding sequences of the three capsid proteins VP2, VP6, and VP7 triggers the synthesis of all three encoded RV proteins. Moreover, these proteins assemble into RVLPs within the transduced cells. When administered intramuscularly, the RV protein encoding HSV-1 amplicon vectors induce a RV specific antibody response detected in sera as well as in milk of vaccinated dams and in addition in sera of their unvaccinated offspring nursed by vaccinated animals. Although lactogenic transfer of RV specific immunoglobulin G (IgG) to the pups of vaccinated dams occurred, full protection of the newborn against RV induced symptoms was not achieved under the present experimental conditions.

## 2. Results

### 2.1. Codon-Optimized Herpes Simplex Virus Type-1 Amplicon Vectors Trigger Synthesis of Rotavirus Structural Proteins

**Figure 1:**
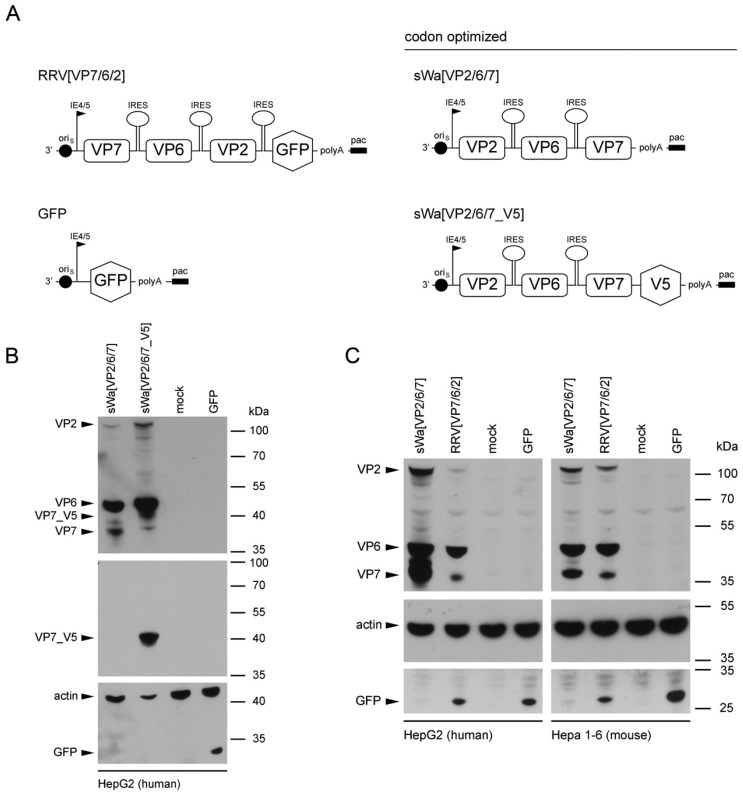

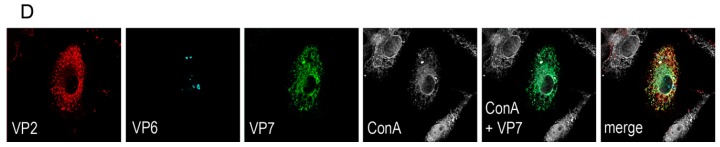
Rotavirus (RV) protein synthesis upon herpes simplex virus type-1 (HSV-1) amplicon vector transduction. (**A**) Schematic representation of the polycistronic gene expression cassettes packaged into herpesvirus amplicon particles. Herpes simplex virus type-1 amplicon particles deliver a DNA cassette comprising a single polycistronic messenger RNA containing the coding sequences of three RV capsid proteins VP2, VP6 and VP7 separated by internal ribosome entry sites (IRES) and controlled by the HSV-1 immediate early (IE) 4/5 promoter. The HSV-1 origin of DNA replication (oriS) and packaging/cleavage signal (pac) as well as the SV40 polyadenylation sequence (polyA) are indicated. The synthetic RV cassettes derived from the human Wa strain were adjusted to human codon usage (synthetic strain Wa, sWa). The RRV[VP7/6/2] was derived from the rhesus strain RRV and encodes, in addition to the RV proteins, for the enhanced green fluorescent protein (EGFP) as reporter gene. The sWa[VP2/6/7_V5] amplicon vector harbors a C-terminal V5 tag at VP7. The GFP amplicon vector encoding EGFP was used as control vector; (**B**) Rotavirus gene expression from codon optimized transgene cassettes. HepG2 cells were transduced with the indicated amplicon vectors (multiplicity of infection, MOI 2) or non-transduced (mock), and total cell lysates were harvested at 24 h post-transduction (hpt). Transgene expression was analyzed by Western blotting using the polyclonal anti-RV antibody to detect RV proteins (upper panel), the anti-V5 antibody for detection of the V5 tagged VP7 protein (middle panel), or the anti-actin and anti-GFP antibodies (lower panel). Detection of actin served as loading control. The predicted migration lengths of VP2 (104 kDa), VP6 (45 kDa), VP7 (37 kDa), VP7_V5 (42 kDa), actin (42 kDa) and GFP (27 kDa) are indicated on the left with arrows. The positions of the molecular weight markers are indicated on the right in kDa; (**C**) Analysis of protein synthesis in human and mouse cells. Human HepG2 or mouse Hepa 1-6 cells were transduced either with sWa[VP2/6/7], RRV[VP7/6/2] or GFP HSV-1 amplicon vectors (MOI 2) or non-transduced (mock) and total cell lysates were harvested 24 hpt. Transgene expression was analyzed using the polyclonal anti-RV antibody for detection of RV proteins (upper panels), the anti-actin antibody (middle panels) or the anti-GFP antibody (lower panels). Detection of actin served as loading control. The predicted migration lengths of the individual proteins are indicated on the left and the positions of the molecular weight markers are shown in kDa on the right; (**D**) Intracellular distribution of HSV-1 amplicon vector encoded RV proteins. Vero2-2 cells were transduced with the amplicon vector sWa[VP2/6/7_V5] at an MOI of 1 and analyzed by immune fluorescence 24 hpt with a Leica SP8 confocal laser scanning microscope. The RV proteins were stained using the following antibodies: anti-VP2 antibody directly labeled with Zenon Alexa-Fluor 594 (red), anti-VP6 and the anti-mouse Alexa-Fluor 633 (cyan), anti-V5 directly labeled with fluorescein isothiocyanate (FITC) for the V5-tagged VP7 (green). The endoplasmic reticulum (ER) was stained using the Alexa-Fluor 405 conjugated lectin concanavalin A (ConA) (grey).

The HSV-1 amplicon vectors used in this study are shown schematically in Figure 1A. The sequence for the construction of the synthetic transgene cassettes of sWa[VP2/6/7] and sWa[VP2/6/7_V5] was derived from the human RV strain Wa, codon-optimized to human gene codon preference and constructed as described in the Materials and Methods section. The HSV-1 amplicon vector encoding the green fluorescent protein (GFP) was used as a control vector.

Synthesis of the RV proteins mediated by the synthetic HSV-1 amplicon vectors sWa[VP2/6/7] and sWa[VP2/6/7_V5] was confirmed by Western blot analysis of total cell lysates following transduction of cells. All three RV proteins VP2, VP6 and VP7 were synthesized in human and non-human cells shown in Figure 1B for the human hepatocellular cells HepG2. Furthermore, the V5 tagged VP7 (VP7_V5) was detected using the V5 antibody in cells transduced with sWa[VP2/6/7_V5] (Figure 1B, middle panel).

The design of both synthetic HSV-1 amplicon vectors sWa[VP2/6/7] and sWa[VP2/6/7_V5] was based on the vector RRV[VP7/6/2] with the aim to improve transcription/translation of the transgene cassette through codon-optimization to human codon usage. Through optimization of the codon usage, the codon adaption index (CAI) increased from 0.605 to 0.895 for human and from 0.606 to 0.892 for mouse. Indeed, the codon-optimized vector sWa[VP2/6/7] induced an enhanced protein synthesis in human cells over the non-codon-optimized vector RRV[VP7/6/2] (Figure 1C, left panel). This effect was not observed in the murine hepatoma cell line Hepa1-6 (Figure 1C, right panel) even though the CAIs for the codon usage of humans and mice were very similar.

Next, the subcellular distribution of the different RV structural proteins in HSV-1 vector transduced Vero2-2 cells was examined by confocal laser scanning microscopy and specific antibodies (Figure 1D). The VP2 signal was detected as punctate pattern distributed throughout the cytoplasm and VP6 expression appeared as discrete punctate and circular structures in the cytoplasm. V5-tagged VP7 was detected in the cytoplasm as dot structures, some forming aggregates. When compared to the concanavalin A (ConA) staining of the endoplasmic reticulum (ER) membranes, VP7 was localized at the ER as previously described [15,21].

These results demonstrated that all three RV structural proteins VP2, VP6, and VP7 were synthesized from the newly designed HSV-1 cassettes in human and non-human cells and the codon-optimization led to an increased quantity of generated RV proteins in human cells.

### 2.2. Ultrastructural Analysis of Intracellular Rotavirus-Like Particles

**Figure 2:**
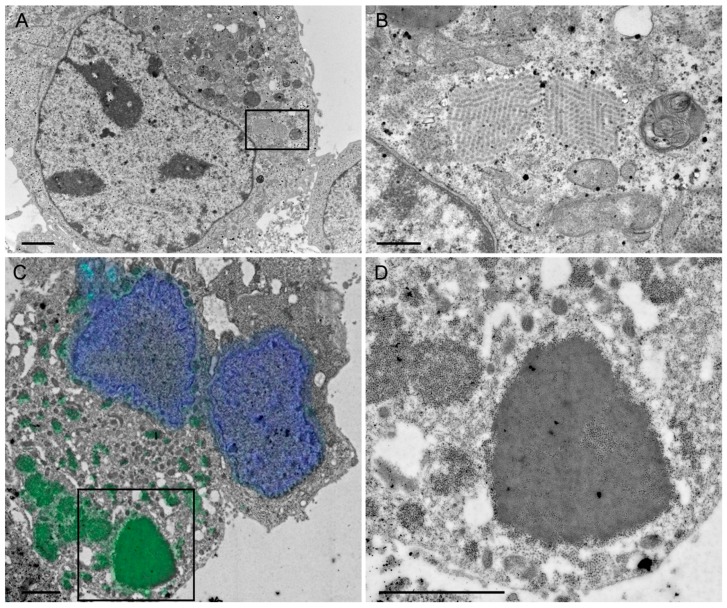
Ultrastructural analysis of transduced cells using electron microscopy. (**A**,**B**) Transmission electron micrographs of RVLP formation in the cytoplasm of vector-transduced cells. HepG2 cells were transduced (multiplicity of infection, MOI 5) with the HSV-1 amplicon vector sWa[VP2/6/7]. The cells were harvested 24 hpt from monolayers, processed as described in the material section and analyzed in a transmission electron microscope equipped with a charge-coupled device (CCD) camera; (**B**) Enlargement of the region marked in (**A**) showed clusters of regular circular structures found in the cytoplasm of the transduced cell. Scale bar: (**A**) 2 µm; (**B**) 0.5 µm. (**C**,**D**) Correlative light and electron microscopy. HepG2 cells were transduced (MOI 5) with the HSV-1 amplicon vector sWa[VP2/6/7_V5]. The cells were harvested 24 hpt from monolayers by pelleting and fixation with 4% formaldehyde and embedded in LR White. Ultrathin sections were directly collected on grids, prepared for immune fluorescence and images were taken using a fluorescence microscope. Thereafter, the same grids were further processed for electron microscopy. The fluorescent image was opened with the software Maps on the scanning electron microscope (SEM) computer, aligned with the secondary electron image and a tileset of images was recorded using the high-angle annular dark-field detector; (**C**) Overlay of the fluorescent and the electron microscope image of a stitched tileset of images covering the entire cell of interest. On the fluorescence micrograph, 4′,6-diamidino-2-phenylindole (DAPI) was used to stain the nuclei (blue), and RV proteins were stained using the anti-RV serum and the secondary antibody conjugated with Alexa-Fluor 488 (green); (**D**) Enlargement of the area of interest of the stitched tileset showing the highly RV-positive-marked electron-dense region marked in (**C**). Scale bar: (**C**,**D**) 1 µm.

In a next step, HepG2 cells were transduced with sWa[VP2/6/7] and ultrathin sections were prepared for transmission electron microscopy (TEM) (Figure 2A,B). The structures detected resembled RV particles or viroplasms [22]. To prove that these structures represented accumulated RV proteins, correlative light and electron microscopy [23] was done (Figure 2C,D). The ultrathin sections of vector-transduced cells were labeled as indicated in the Materials and Methods section and imaged with a fluorescence microscope. The resulting micrograph was used as a map to record the same area in the electron microscope (EM). Figure 2C shows an overlay of the fluorescent and the EM images and Figure 2D is an enlargement of the area of interest recorded by the EM, focusing on the highly RV-antibody-positive marked and electron-dense regions. Such electron-dense and antibody-positive regions showed regularly shaped circular structures that formed aggregates or clusters resembling virus particles. 

We conclude that the observed circular structures represent indeed RVLPs formed in the cytoplasm of HSV-1 transduced cells resembling viroplasms found in RV-infected cells.

### 2.3. Rotavirus Specific Antibody Response Induced by Immunization with sWa[VP2/6/7]

**Figure 3:**
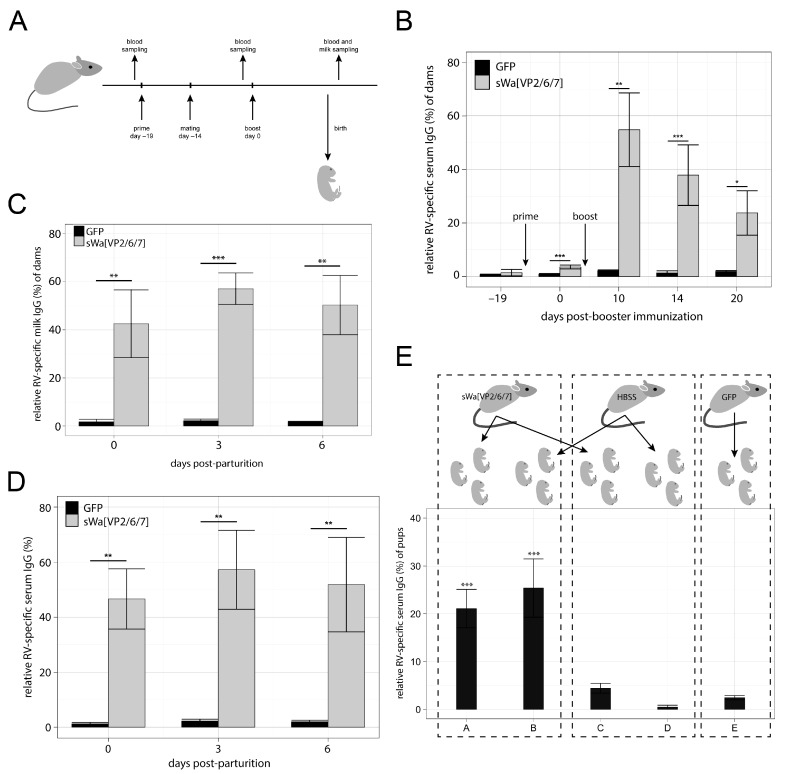
Immune response in mice upon immunization with the vaccine vector sWa[VP2/6/7]. (**A**) Schematically represented in vivo studies as timeline. Before the immunization of female BALB/c mice a blood sample was collected from each animal. Five days after intra muscular (i.m.) injection of the prime dose, a male was added for mating. Before booster immunization, blood samples were taken. For the first set of experiments, mice received a booster immunization at 19 days after prime dose injection, and, for the second set of experiments, at 20 days after prime dose injection. Blood and milk samples were taken on a regular basis starting on the day of parturition. (**B**–**D**) Rotavirus specific immunoglobulin G (IgG) serum (**B**,**D**) or milk (**C**) levels of immunized mouse dams determined by enzyme-linked immunosorbent assay (ELISA). Rotavirus-specific IgG in sera (1:5000 dilution) or milk (1:500 dilution) of either sWa[VP2/6/7] or GFP amplicon vector immunized animals was measured and plotted relative to the positive control applied on each ELISA plate that was taken as 100%. As positive control always the same sera of a RV hyperimmunized mouse from a previous study [17] (diluted 1:5000) was used. Relative IgG values were plotted on the ordinate in relation to the time after booster immunization in days on the abscissa. Values from naïve sera are plotted on the time point −19 as these samples were taken before the first immunization (**B**). In (**B**), time point 0 indicates relative sera IgG levels before booster immunization, time point 10 includes values from animals from 9 to 10 days post-booster immunization (dpb), time point 14 includes samples taken on 13 and 14 dpb and time point 20 from 20 to 21 dpb. Standard deviations between the measurements from sera of individual animals are indicated. In (**C**,**D**) time point 0 indicates values from milk (**C**) or serum (**D**) samples taken on the day of parturition, time point 3 includes all milk samples from three or four days after parturition and time point 6 includes all samples collected six or seven days post-delivery. Standard deviations between measurements from milk of individual animals are indicated. *p*-values between the sWa[VP2/6/7] and GFP immunized groups are indicated: *, *p* < 0.05; **, *p* < 0.01; ***, *p* < 0.001. (**E**) Rotavirus-specific IgG levels in pup sera determined by ELISA. Rotavirus-specific IgG in terminal sera from the offspring of either sWa[VP2/6/7] or GFP amplicon vector immunized dams or Hanks' balanced salt solution (HBSS) injected control dams was plotted in relation to the positive control (set as 100%). In group A, offspring was born and raised by sWa[VP2/6/7] immunized dams. In group B, offspring was born from HBSS injected dams but raised by sWa[VP2/6/7] immunized dams. Group C includes offspring born from sWa[VP2/6/7] immunized dams and raised by HBSS injected control dams. Group D includes offspring born and raised by HBSS-injected control animals. Group E includes offspring born and raised by GFP vector immunized dams. Here, dams obtained the booster dose 20 days after prime immunization. The standard deviation of each group is indicated. *p*-values of A versus C, D or E or B versus C, D or E are indicated: ***, *p* < 0.001.

We injected female mice with 10^6^ transducing units (TU) per dose intramuscularly (i.m.) in a prime-boost regimen with sWa[VP2/6/7] or the GFP encoding amplicon vector as control. Five days after the first immunization, males were added to the females for mating (Figure 3A). Nineteen days after the prime immunization, dams were boosted. Blood and milk samples from dams were collected after parturition and tested for RV specific IgG antibodies using a RV (strain Wa) specific enzyme-linked immunosorbent assay (ELISA). Rotavirus specific IgG antibodies were found in sera of all sWa[VP2/6/7] immunized mice that differed significantly from the sera of GFP immunized control mice (Figure 3B). The data used to generate the plot in 3B as well as the number of animals included in this study and *p*-values are provided in the Appendix A as Table A1. Mice immunized with sWa[VP2/6/7] showed a relative RV-specific IgG (rIgG) level of 54.85% at day 10 post-booster immunization (dpb), which decreased to 37.89% rIgG at 14 dpb and then decreased to 23.78% rIgG at 20 dpb. Relative IgG of pre-immune sera from animals of both groups was close to 0 (rIgG value of 1.45% for animals of the sWa[VP2/6/7] group and 0.69% in animals of the GFP group). The rIgG levels of the GFP vector immunized control group were low over the entire duration of the experiments with 2.15% at 10 dpb, 1.38% at 14 dpb and 1.92% at 20 dpb. This indicates that a RV specific antibody response was triggered upon sWa[VP2/6/7] vector immunization.

Rotavirus-specific IgG in milk of sWa[VP2/6/7] immunized mice paralleled the titer in their sera (Figure 3C,D; for details see Appendix A in Table A2 and Table A3). On the day of parturition (day 0), levels in milk from sWa[VP2/6/7] immunized animals was found to be at 46.64%, increased to 57.26% on day 3 and slightly decreased to 51.83% rIgG on day 6 after parturition. In contrast, GFP immunized animals showed background IgG in milk with 1.24% on day 0, 2.25% on day 3 and 1.97% on day 6 after parturition. Levels of RV-specific IgA antibody in all of the milk or sera samples tested were below the sensitivity of the ELISA used. Importantly, titer of sera and milk antibodies were similar between delivery and the first seven days (Figure 3C,D, Table A2 and Table A3).

We further analyzed the isotypes of RV specific IgG raised upon sWa[VP2/6/7] immunization and GFP controls (Table A4). All sWa[VP2/6/7] immunized dams showed higher levels of IgG2a when compared to IgG1 (Table A5). Sera of their offspring had comparable ratios of IgG1/IgG2a as the according mothers (Table A5) indicating a type 1 T helper (Th1) immune response induced by vaccination.

In a next experiment dams were immunized in a prime-boost regimen with either sWa[VP2/6/7] amplicon or as control GFP amplicon or Hanks’ balanced salt solution (HBSS) (Figure 3A; Table A6 and Table A7 for milk and serum rIgG levels of dams used for this experiment), but pups were swapped after birth as shown in Figure 3E. A similar RV specific IgG titer was found in terminal sera of pups from sWa[VP2/6/7] immunized dams as in sera from pups nursed from HBSS control injected dams (Figure 3E, group A, B). The RV specific IgG of the sWa[VP2/6/7] immunized dams was thus passed to their offspring or the nursed control pups by the lactogenic route. We also exchanged the offspring from sWa[VP2/6/7] immunized dams with the offspring of HBSS injected control dams immediately after birth. Because of the terminal exsanguination, only the remaining maternal antibody titer of the pups after six days following the transfer to the control dam could be analyzed (Figure 3E, group “C”). This antibody titer was still elevated compared to the controls (Figure 3E, group “D”). All data to generate the plot in Figure 3E and the number of included animals for this experiment are shown in Table A8 in the Appendix A. Table A9 in the Appendix A shows *p*-values between all groups in Figure 3E.

We conclude, that RV specific IgG antibody transfer via the placental route occurred. Moreover, the level of antibody had to be maintained by the lactogenic route by immunized dams or substituted for naïve pups by milk from immunized dams (Figure 3E). In conclusion, we were able to show that injection of the sWa[VP2/6/7] amplicon vector raises a RV specific immune response, detected as IgG in sera as well as in milk of immunized animals and that this response is passed to the offspring via both the placental and the lactogenic route.

### 2.4. Protective Effect of Maternal Antibodies against Rotavirus Challenge

We further tested if the vaccine-induced immune response in the suckling mice was sufficient to protect them from the symptoms of RV infection. Therefore, we challenged the pups of all groups (either swapped or non-swapped) with oral gavage of mouse RV—strain EDIM, 10× diarrhea dose 50 (DD50)—at two to five days after birth. All pups developed diarrhea within comparable periods of incubation time. Thus, the immune response raised was not sufficient to protect the suckling mice from RV induced symptoms in our experimental settings.

It has been reported that RV can become systemic leaving the intestinal route and emerging into the blood [24,25]. As the strain EDIM cannot be propagated in vitro [26], the viral load in the sera of pups was analyzed using a RV specific quantitative real-time polymerase chain reaction (RT-qPCR). The first set of experiments showed promising results indicating that pups from vaccinated dams had lowest DD50 (DD50/mL) titers whereas pups swapped had 20–50 times higher DD50/mL values that reflected the antibody titer (Table A10). Subsequent experiments indicated variables due to the age of the pups at the time of challenge and thus possible developmental stage of immune maturation, weight of the pups and limitation of sera collection due to the need to kill the experimental animals (Table A11). We concluded that it was ethically questionable to control all these variables by selecting a multitude of animals in order to further standardize immunization including controls, birth date, size of the litter and body weight of the pups for the various swap experiments.

### 2.5. Serum Antibodies Raised in Vaccinated Dams were Specific for Rotavirus Proteins VP2 and VP6

**Figure 4:**
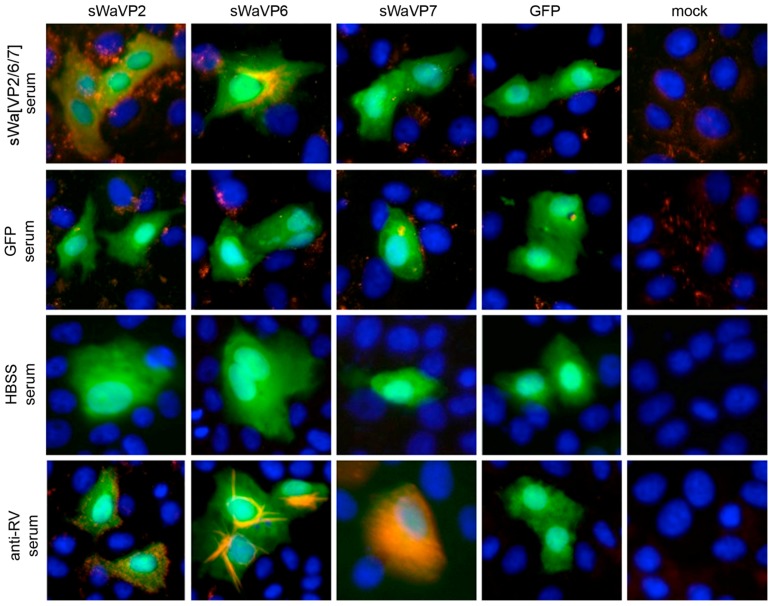
Screening of mice sera using immune fluorescence show VP2- and VP6-specific antibodies in the sera of vaccinated dams. Vero2-2 cells transduced (MOI 1) with the corresponding monocistronic amplicon vectors encoding individual RV proteins VP2, VP6 or VP7 were stained 24 hpt by immune fluorescence. The sera of vaccinated dams served as primary antibodies (diluted 1:100) and were detected by the secondary antibody anti-mouse conjugated with Alexa-Fluor 594 (red). GFP fluorescence (green) expressed as reporter gene from each amplicon vector served to identify transduced cells, DAPI was used to stain the nuclei (blue). Polyclonal anti-RV serum served as positive control.

During our challenge experiments, protection against an oral RV challenge of the suckling mice was not achieved. In a next step, we analyzed the specificity of the RV antibodies in sera of vaccinated dams. Our immunization vector sWa[VP2/6/7] comprised three RV antigens, the main structural proteins VP2, VP6 and VP7. We thus expected antibodies against all three RV antigens in the immunized animals. To test this, immune fluorescence was performed using the sera from vaccinated mice and appropriate controls (Figure 4). To generate each antigen, Vero2-2 cells were transduced with amplicon vectors encoding each of the single RV protein or GFP sera of dams vaccinated with sWa[VP2/6/7] bound VP2 and VP6, but not VP7 (Figure 4, first row). This was true for all mice of this group. Sera of dams vaccinated with the control vector only expressing GFP and of HBSS control dams did not recognize any of the RV proteins specifically (Figure 4, middle rows), although some very minor unspecific background staining was seen occasionally. In summary, only antibodies against the RV VP2 and the VP6 proteins were detected in sera of the sWa[VP2/6/7] vaccinated dams.

### 2.6. Composition of Rotavirus-Like Particles Produced in Herpes Simplex Virus Type-1 Transduced Cells

**Figure 5:**
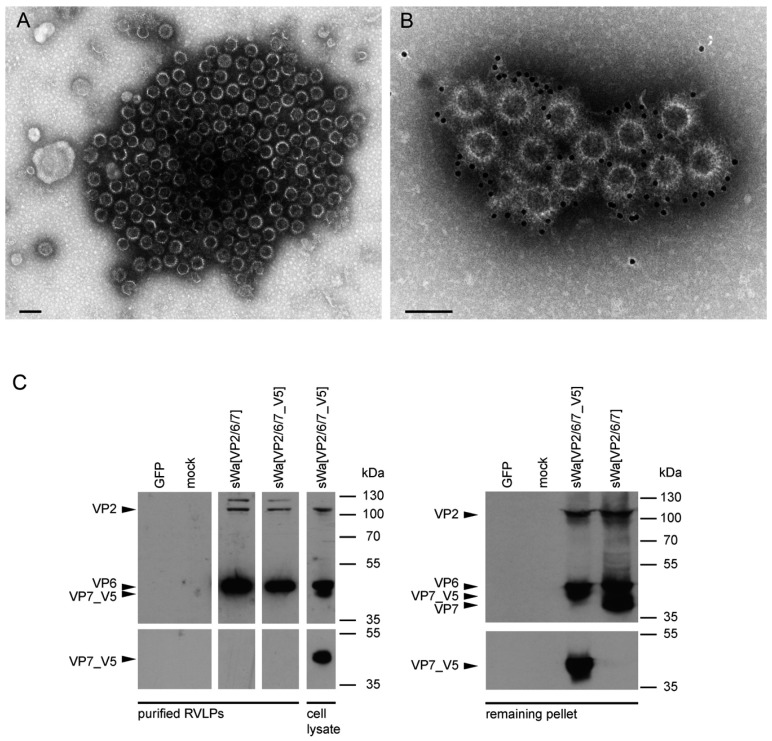
Analysis of rotavirus-like particles (RVLPs) produced in HSV-1 amplicon transduced cells. (**A**,**B**) electron micrographs of HSV-1 amplicon vector encoded RVLPs. HepG2 cells were infected with the polycistronic HSV-1 amplicon vector sWa[VP2/6/7] and two days after transduction, RVLPs were purified over a sucrose cushion and the concentrated particles were analyzed by transmission electron microscopy. (**A**) Negative staining of RVLPs from HepG2 cells transduced with sWa[VP2/6/7]. (**B**) Immunogold staining of the same sample of RVLPs as in (**A**) using a polyclonal anti-RV serum as primary antibody and a secondary antibody coupled to 12 nm colloidal gold particles. Scale bar: 100 nm (**A**,**B**). (**C**) Double-layered purified RVLPs and remaining RV proteins in the cell pellet. Vero2-2 cells were transduced with the indicated HSV-1 amplicon vectors (MOI 2) or non-transduced (mock). Cells were harvested 48 hpt and RVLPs were purified and concentrated from the supernatant over a sucrose cushion. The concentrated RVLPs (purified RVLPs, left panel) and the RV proteins remaining in the cell pellet obtained during RVLP harvesting (remaining pellet, right panel) was analyzed by Western blotting using a polyclonal anti-RV antibody for detection of RV proteins or the monoclonal anti-V5 antibody to detect V5 tagged VP7. In the left panel, whole cell lysate of sWa[VP2/6/7_V5] transduced (MOI 2) Vero2-2 cells was taken as positive control. The predicted migration lengths of the RV proteins are indicated on the left with an arrow and the positions of the molecular weight markers are indicated on the right in kDa.

Previous work showed that VP7 might play a key role in passive protection of pups from RV-induced diarrhea [27]. Based on the results from the immune fluorescence analysis of the mice sera, we were able to raise VP2- and VP6-specific antibodies in the blood of sWa[VP2/6/7] vaccinated dams, but no VP7-specific antibodies. The use of HSV-1 amplicon vectors to launch the in situ production of RVLPs provides the advantage that as opposed to injection of purified VLPs, the delivery of VLP encoding genes into host cells results in the intracellular production of antigens. In our case, we expected the formation of triple-layered RVLPs. Therefore, we further characterized the nature of our antigen used for vaccination. In a first step, RVLPs formed upon transduction of sWa[VP2/6/7] gene expression cassettes was analyzed using electron microscopy (Figure 5A,B) and in a second step, the composition of purified particles was examined using Western blot analysis (Figure 5C). Negative staining transmission electron microscopy of the purified cell lysates from sWa[VP2/6/7] transduced cells showed that the synthesized RV structural proteins assembled into RVLPs similar in size and structure to wild-type RV particles [28]. Further immunogold labeling of these RVLPs using the rabbit polyclonal anti-RV serum and a secondary antibody conjugated to gold particles confirmed the VLP’s identity as RV-like.

To analyze the composition of the isolated RVLPs, Western blot analysis of the purified RVLPs was performed (Figure 5C). This indicated that the observed RVLPs consisted predominantly of the two inner layers VP2 and VP6 (Figure 5C, left panel), whereas the outer layer protein VP7 remained in the cell debris pellet obtained during the purification of the RVLPs (Figure 5C, right panel). Moreover, VP7 is a transmembrane glycoprotein localized at the ER [29] and seemed to localize with the ER membranes (Figure 1D) even though VP2 and VP6 were co-expressed. In summary, even though VP7 was abundantly present in transduced cells (Figure 1B,C), it was hardly incorporated into the isolated RVLPs leading to the conclusion that in our system double-layered RVLPs were preferentially formed.

## 3. Discussion

The salient new findings in our study include: (1) transduction of cells with our HSV-1 amplicon vector delivering a DNA cassette encoding a single polycistronic messenger RNA, comprising the codon-optimized sequences of the three RV capsid proteins VP2, VP6, and VP7, triggered the synthesis of all three encoded RV proteins; (2) cell-type dependent increase of the desired protein synthesis due to codon-optimization of the capsid protein coding sequences in the vector; (3) assembly of VP2 and VP6 into double-layered virus-like particles, while VP7 was synthesized in the same cell but, apparently, excluded from particle formation; (4) intramuscular inoculation of these amplicon particles resulted in the production of RV-specific antibodies against VP2 and VP6 but, interestingly, not VP7; (5) these antibodies were transferred to mouse progeny via the uterine and lactogenic pathway; and (6) despite efficient antibody transfer to offspring, clinical protection against diarrhea upon experimental infection of the pups with a heterologous, mouse-specific RV was not achieved.

Purification and propagation of RV from clinical fecal specimen in cell culture is difficult and adaptation to growth in continuous cell lines at high titers usually requires multiple rounds of passaging in primary cells [30]. Consequently, the results of adaptation usually include alterations in the amino acid sequences of the proteins of immunogenic interest. We solved this problem by using a synthetic and codon-optimized DNA cassette, which maintained the original (RV strain Wa, Dhaka isolate) amino acid sequences of the three major capsid proteins VP2, VP6, and VP7. The particular strengths of this approach are three-fold: (1) the vaccine can easily be adjusted to the circulating RV strain, since only the packaged DNA sequence needs to be modified; (2) HSV-1 amplicon vectors have a high transgene capacity (up to 150 kb) that ensures that multiple copies of the RV protein encoding genes are delivered and thereby trigger a high amount of protein synthesis; and (3) VLP purification, which is very expensive and time consuming, can be omitted because VLPs are being formed in situ.

A rationale of structural vaccinology is to maintain the native three-dimensional structure and to stabilize the conformation of antigens to induce an efficacious immune response. We and others [14,17,27,31] reported that assembly of structural proteins into VLPs might be crucial for protection against RV infections. The viral structure is assembled by several proteins on the VLP surface mimicking the native virions and allowing crucial overlapping epitopes to be recognized by antibodies that would not be possible by the use of single individual subunits as antigens. Adopting these principles, we propose that the structural aspect of RVLPs is important for RV specific generation of protective antibodies. Based on electron microscopic analysis, the RVLPs produced in vitro were highly analogous to native RV particles. However, further analysis revealed that cell culture purified RVLPs were predominantly composed of VP2 and VP6 but excluded VP7 (Figure 5C). As known from literature [1,32], and shown by our data of the intracellular localization of the synthetized proteins (Figure 1D), VP7 associates with the membranes of the ER, whereas VP2 and VP6 form punctate structures within the cytoplasm but do not co-localize with the ER membranes. It is not fully understood how triple-layered particles (TLP) can form under these conditions. However, Coste et al. were able to purify TLPs following co-infection of insect cells with three different baculoviruses [27] (i.e., one for the expression of each of the three capsid proteins, VP2, VP6, and VP7).

In contrast to these findings and upon synthesis in mammalian cells, VP7 was abundantly synthesized but did not co-purify with the VLPs. Instead, upon VLP purification, VP7 remained in the pellet, together with the cellular debris. Even more surprisingly, antibodies against VP7 were not induced under these circumstances. Consequently, it might be that the requirements for TLP formation are different for insect cells and mammalian cells. Indeed, according to others, co-expression of NSP4 and/or VP4 together with the three capsid proteins may play a pivotal role in the assembly of the third layer [1]. A better understanding of the RV assembly process is needed to solve the problem of VP7 incorporation into VLPs in the future.

Protection against RV infection in adult mice can be measured by reduction of fecal virus shedding after oral challenge (adult mouse model). However, in all species, including humans and mice, RV infection in adults is usually asymptomatic and does not cause diarrhea. Similar to human babies and in contrast to adult humans and mice, newborn mice develop severe diarrhea upon RV infection, particularly during the first 14 days of their life [33]. However, the immune system of newborns is not fully mature at this stage. Thus, immunization of mothers, resulting in the protective transfer of their antibody repertoire to the offspring, represents an important alternative that can also be mimicked in the mouse model. Indeed, protection against RV diarrhea by passive maternal transfer of antibodies has been reported (mouse maternal antibody model) using live-rotavirus vaccines [34], recombinant adenovirus expressing VP7 [35], recombinant VP6 and VP8 proteins expressed in *E. coli* [36], VLPs assembled in insect cells [27] or capsid proteins VP2 and VP6 expressed in *E. coli* and assembled into VLPs post-purification [14].

It is widely accepted that the B-cell arm of the immune response plays a major role in controlling RV infection. Observations are reported not only from mouse experiments but also from clinical studies in piglets and children [37,38,39]. However, the mechanisms by which antibody producing cells precisely act against RV are unclear and therefore extensively discussed in the field. It is important to note that the amplicon vector induced a Th1 type of immune response with an increased level of IgG2a over IgG1 (Table A4 and Table A5) believed to be effective for the control of viral infection. This Th1 response might be an indication that our system would be able to induce mucosal IgA in humans.

In the adult mouse model, fecal IgA antibodies against VP6 have been the main correlate for protection against RV infection and it has been suggested that VP6 specific antibodies are taken up by the polymeric Ig receptor of the infected enterocyte neutralizing RV intracellularly [40]. However, the passive transfer of VP6-specific antibodies to newborns was not sufficient to protect against diarrhea [27,31,41]. Coste et al. demonstrated that nasal immunization of mice with purified TLPs assembled in insect cells and comprising VP2, VP6, and VP7 triggered high milk and serum antibody titers. Furthermore, milk but not serum antibodies were associated to the protection of suckling mice and that antibodies to VP7 played a crucial role [27].

Having the same antigens (VP2, VP6, and VP7) included in our approach and having shown that all three proteins were being synthesized in transduced cells, we were confident to achieve similar results in a protection study as Coste et al. [27], though with IgG and upon in situ synthesis of the viral proteins instead of immunizing with purified VLPs. Indeed, our vaccinated mice produced high anti RV IgG antibody titers, both in serum and in milk, and they transferred these antibodies to their offspring. However, we were unable to detect any RV specific IgA antibodies in the vaccinated mice. Consequently, a clear protection against diarrhea was not achieved. This was in agreement with previous studies [34,36], which reported that protection from rotavirus diarrhea relied on the lactogenic transfer of IgA against VP7 or VP4. Of course, several factors contribute to clinical protection against RV, including a close match of antibodies against the targeted antigens, the amount and isotype of antibodies as well as the location, where they should act. In the present case, the transfer of antibodies from the immunized mothers to their suckling mice seemed efficient. However, at least three factors might have influenced the protective outcome in a negative way: (1) The stringent stop criteria for the animal experiments did not allow the examination of the duration and severity of RV symptoms in offspring in the different experimental groups; (2) The antibody isotype generated upon vaccination was predominantly IgG, whereas IgA is supposed to provide a better level of protection, particularly in the gut. On the other hand, one may consider that the maternal antibodies had been recovered from the gut as a consequence of suckling milk containing these antibodies. Thus, the challenging virus and the potentially protective antibodies had opportunities to meet and to react in the gut, at least for a certain amount of time. It was, therefore, surprising to note that no obvious protective effect could be achieved. Of note, we used heterologous RVLPs based on the human RV strain Wa and mice were challenged with a heterotypic RV, the murine strain EDIM. Thus, it might be that even if VP7-specific antibodies were raised, no protection was observed because of the poor cross-reactivity between the raised antibodies; (3) According to Butler et al. [21], the mammals cluster into three groups, with respect to lactogenic immunity. Rodents, classified in group 2, show particularly good absorption of IgG from the gut, which drains the gut from functional antibodies. Humans, classified in group 1, absorb only little immunoglobulins from the gut, leaving the antibodies to work where they are supposed to throughout a RV infection. Pigs, classified in group 3, show extensive absorption of all classes of immunoglobulins but only for the first 12 h. Afterwards, the milk antibodies are known to work for several weeks particularly well in the porcine gut. This property is even enhanced by the ability of the pigs to produce high amounts of IgA in response to vaccination. 

We conclude from the reasons stated above that the presently used mouse model may be suboptimal for testing RV vaccines designed for human use. Two possible future approaches emerge from these considerations: (1) The vaccine should be tested in pregnant women in order to analyze the antibody specificities and isotypes in their serum as well as their milk; (2) Pigs or cattle, which also belong to group 3, might be vaccinated in order to produce decent amounts of IgA against various RV strains. These IgA may find use in medicine as well as in veterinary medicine.

## 4. Materials and Methods

### 4.1. Cells and Viruses

Vero 2-2 (African green monkey kidney epithelium cells, [42]), Hepa 1-6 (mouse epithelial hepatocytes, ATCC), MA104 (embryonic African green monkey kidney, ATCC) and HepG2 (human liver hepatocellular cells, ATCC) cells were maintained in Dulbecco’s modified Eagle’s medium (DMEM) supplemented with 10% fetal bovine serum (FBS), 100 units/mL of penicillin G, 100 µg/mL of streptomycin, 0.25 µg/mL of amphotericin B and for Vero2-2 cells also with 500 µg/mL of G418 (Thermo Fisher Scientific, Waltham, MA, USA). The murine wild type (wt) RV strain EDIM was obtained from Harry Greenberg (Department of Medicine and Microbiology and Immunology, Stanford University School of Medicine, Stanford, CA, USA). The titration of wt RV EDIM used to challenge mice after vaccination was described previously [26]. The human RV strain Wa was obtained from Catherine Eichwald (University of Zurich, Zurich, Switzerland); it was propagated in MA104 cells as previously described [43]. 

### 4.2. Construction of Herpes Simplex Virus Type-1 Amplicon Plasmids

The sequence for the construction of the synthetic transgene cassettes of sWa[VP2/6/7] and sWa[VP2/6/7_V5] was derived from the human RV strain Wa (Dhaka isolate) and codon-optimized to human gene codon preference—verified by Genescript (Piscataway, NJ, USA) and synthesized by Biomatik (Cambridge, Ontario, Canada)—and predicted splice sites were removed [44]. The codon adaption index [45] was calculated using The European Molecular Biology Open Software Suite (EMBOSS) [46]. Herpes simplex virus type-1 amplicon plasmids were cloned using Gateway technology (Thermo Fisher Scientific, Waltham, MA, USA). The attB flanked synthetic gene expression cassettes (sWaRV) encoding the RV proteins VP2, VP6 and VP7, separated by internal ribosome entry sites (IRES) either with or without stop codon at the 5′ end were generated by Biomatik. The Gateway B/P recombination between the attB flanked sWaRV cassette and the attP containing donor vector pDONR221 led to two entry plasmids containing the sWaRV gene expression cassette flanked by attL sites either with (pE_sWaRV_STOP) or without stop codon (pE_sWaRV). The amplicon plasmid used to produce sWa[VP2/6/7] vector stocks was generated by the Gateway L/R recombination between the attL sites containing entry vector pE_sWaRV_STOP and the attR sites containing destination vector pHSV-EYFP-RfC_C.1. For production of sWa[VP2/6/7_V5] amplicon vector stocks, pE_sWaRV was recombined with the destination vector pHSV-V5/His. The amplicon expression plasmids pHSV-EYFP-RfC_C.1 and pHSV-V5/His contain a transcription unit consisting of the HSV-1 immediate early (IE) 4/5 promoter and the SV40 polyadenylation signal as well the HSV-1 origin of replication (oriS) and the HSV-1 packaging/cleavage signal (pac) necessary for packaging into helper virus-free HSV-1 amplicon particles. The HSV-1 amplicon vectors encoding single structural RV proteins have been generated as follows: The single RV genes were amplified using the synthetic gene expression cassettes (sWaRV) as the template. The resulting PCR product was inserted into pHSV_S_ [47]. The resulting HSV-1 amplicon plasmids encode for a single RV protein, sWaVP2, sWaVP6 or sWaVP7; and after an IRES, the EGFP to identify vector-transduced cells.

### 4.3. Production of Herpes Simplex Virus Type-1 Amplicon Vector Stocks

Helper virus-free HSV-1 amplicon vector stocks were prepared as previously described [16,26]. Briefly, Vero 2-2 cells were co-transfected with amplicon plasmid DNA, the fHSVΔpacΔICP27 BAC DNA, and plasmid pEBHICP27 using Lipofectamine LTX and Plus Reagent (Thermo Fisher Scientific). After 72 h, cells were scraped into the medium, freeze/thawed, sonicated, and the cell debris was removed by centrifugation. For immunization of mice, vector stocks were further purified and concentrated by centrifugation over a 25% sucrose cushion. For titration, Vero 2-2 cells were infected with the amplicon vectors, and after 24 h stained for immune fluorescence using the appropriate antibodies and fluorescent cells were counted using an inverted fluorescence microscope (Axio Observer inverted microscope, Zeiss AG, Oberkochen, Germany). The titers were determined as TU/mL.

### 4.4. Rotavirus-Like Particles Purification

HepG2 or Vero2-2 cells were transduced (MOI 2) with the indicated HSV-1 amplicon vector and RVLPs were harvested 48 hpt as described previously [48,49]. Briefly, cells were scraped into the medium and cell membranes were disrupted by repeated cycles of thawing/freezing. The cell debris was removed by centrifugation at 1400× *g* and filtration through a 0.45 µm filter. The cleared supernatant was loaded onto a 10% sucrose cushion and concentrated at 100,000× *g* for 2 h at 16 °C. For protection, protease inhibitor (protease inhibitor cocktail tablets complete, mini, ethylenediaminetetraacetic acid (EDTA)-free, 1 tablet per 10 mL, Roche Diagnostics, Mannheim, Germany) was added to the supernatant.

### 4.5. Western Blot Analysis

Cells were transduced with the indicated amplicon vectors (MOI 2) and total cell lysates were harvested 24 hpt or 48 hpt if RVLPs were purified. Either whole cell lysates or sucrose purified RVLPs (see section “RVLP purification”) were separated on 10% sodium dodecyl sulfate (SDS)-polyacrylamide gels, transferred to nitrocellulose membranes, probed with primary antibodies, and stained using anti-mouse (Sigma-Aldrich, Buchs, Switzerland) or anti-rabbit (Southern Biotech, Birmingham, AL, USA) IgG antibodies conjugated with horseradish peroxidase (HRP), followed by detection with WesternBright ECL spray (Advansta, Menlo Park, CA, USA) according to the manufacturer’s instructions. Rabbit anti-rotavirus polyclonal serum raised against whole virus (1:4000, strain RF, provided by Didier Poncet, The French National Center for Scientific Research (CNRS)/ National Institute of Agricultural Research (INRA), Gif-sur-Yvette, France), guinea-pig anti-rotavirus polyclonal serum raised against whole virus (1:2000, provided by Catherine Eichwald, University of Zurich, Zurich, Switzerland), mouse anti-GFP monoclonal antibody (1:8000, JL-8, Santa Cruz, CA, USA), mouse anti-V5 monoclonal antibody (1:5000, Molecular Probes, Thermo Fisher Scientific, Waltham, MA, USA) and mouse anti-actin monoclonal antibody (1:10,000, Sigma–Aldrich) were used as primary antibodies. For antibody stripping, membranes were incubated for 15 min with Stripping Buffer (Thermo Scientific, Rockford, IL, USA) and washed three times with phosphate-buffered saline (PBS).

### 4.6. Immunofluorescence

Vero 2-2 cells were grown on 12 mm coverslips (0.17 mm thick) and transduced with the indicated HSV-1 amplicon vector at the specified MOI. The cells were fixed 24 hpt with 3.7% formaldehyde in PBS and treated with 0.1 M glycine in PBS. After permeabilization with PBS containing 0.2% Triton X-100 (PBS-T), the cells were blocked with PBS supplemented with 3% bovine serum albumin (PBS-BSA; Sigma-Aldrich, Buchs, Switzerland). Cells were incubated with the corresponding antibodies diluted in PBS-BSA: anti-VP2 antibody (1:200; provided by Didier Poncet, CNRS/INRA, Gif-sur-Yvette, France) directly labeled with Zenon Alexa-Fluor 594 according to the manufacturer’s instructions (Molecular Probes, Thermo Fisher Scientific), mouse monoclonal anti-VP6 (1:500; Novus Biologicals, Cambridge, UK) and the anti-mouse Alexa-Fluor 633 (1:500; Molecular Probes, Thermo Fisher Scientific), mouse monoclonal anti-V5 antibody directly labeled with fluorescein isothiocyanate (FITC) (1:500; Molecular Probes) for the V5-tagged VP7. The ER was stained using the Alexa-Fluor 405 conjugated lectin ConA conjugated with Alexa Fluor 594 (20 µg/µL in PBS; Molecular Probes). Cells were incubated with 4',6-diamidino-2-phenylindole (DAPI) (1 µg/mL in PBS, Roche, Basel, Switzerland) to visualize nuclei. After washing the cells with PBS and H_2_O, the coverslips were mounted in ProLong Gold (Molecular Probes). Samples were analyzed using a confocal laser-scanning microscope SP8 (Leica Microsystems, Wetzlar, Germany, 63× oil objective (numerical aperture (NA) = 1.40)). For analysis of RV protein-specific antibodies in the sera from immunized mice, sera was diluted 1:100 and detected by staining with a secondary anti-mouse antibody conjugated with Alexa-Fluor 594 (1:500; Molecular Probes). Pictures were taken using the fluorescence microscope (Axio Observer inverted microscope, Carl Zeiss AG, Oberkochen, Germany).

### 4.7. Transmission Electron Microscopy 

#### 4.7.1. Negative Staining of Purified Rotavirus-Like Particles

For negative staining, samples (see section “RVLP purification”) were adsorbed to carbon-coated parlodion films mounted on 300 mesh/inch copper grids (Electron Microscopy Sciences (EMS), Fort Washington, PA, USA) for 10 min washed once with H_2_O, and stained with 2% phosphotungstic acid (PTA), pH 7.0 (Aldrich, Steinheim, Germany) for 1 min. Specimens were analyzed in a transmission electron microscope (CM12, Philips, Eindhoven, The Netherlands) equipped with a CCD camera (Ultrascan 1000, Gatan, Pleasanton, CA, USA) at an acceleration voltage of 100 kV.

#### 4.7.2. Immunogold Labeling of Purified Rotavirus-Like Particles

For immune electron microscopy, samples were adsorbed to carbon-coated parlodion films mounted on 300 mesh/inch copper grids (EMS) for 10 min, blocked with PBS containing 0.1% BSA (PBS-BSA/0.1%) for 10 min, incubated with the polyclonal rabbit anti-RV serum (strain RF, provided by Didier Poncet, CNRS/INRA, Gif-sur-Yvette, France) at a dilution of 1:1000 PBS-BSA/0.1% for 1 h, washed several times with PBS-BSA/0.1%, incubated with goat anti-rabbit IgG coupled to 12 nm colloidal gold particles (Jackson ImmunoResearch, West Grove, PA, USA), washed several times with PBS and H_2_O, and stained with 2% PTA, pH 7.0 (Aldrich) for 1 min. Specimens were analyzed in a transmission electron microscope (CM12, Philips) equipped with a CCD camera (Ultrascan 1000, Gatan) at an acceleration voltage of 100 keV.

#### 4.7.3. Chemical Fixation and Embedding in Epon

HepG2 cells were transduced (MOI 5) with the HSV-1 amplicon vector sWa[VP2/6/7]. After 24 h, the cells were scraped into the medium and resuspended in 2.5% glutaraldehyde (GA), centrifuged for 20 min at 4000 *g* and the pellet was embedded in Epon according to a standard protocol previously described [50]. Briefly, the cell pellet was fixed with 2.5% GA and postfixed with 1% osmium tetroxide. Before embedding in epoxy resin (Epon), the cell pellets were dehydrated through series of solvents. Finally, ultrathin sections were stained with uranyl acetate and lead citrate and coated with carbon. Specimens were analyzed in a transmission electron microscope (CM12, Philips) equipped with a CCD camera (Orius SC1000W, Gatan) at an acceleration voltage of 100 keV. 

### 4.8. Correlative Light and Electron Microscopy

#### 4.8.1. Chemical Fixation and Embedding in LR White

HepG2 cells were transduced (MOI 5) with the HSV-1 amplicon vector sWa[VP2/6/7_V5] and 24 hpt, the cells were scraped into the medium and centrifuged for 20 min at 4000× *g*. The resulting cell pellet was fixed with 4% formaldehyde in 0.1 M Na/K phosphate buffer for 4 h. Thereafter, the pellet was dehydrated with ascending ethanol series starting at 70%, followed by 80%, 96% and three times in absolute ethanol for 10 min each. Next, the pellet was incubated at 4 °C for 1.5 h in a 2:1 mix of LR White/Ethanol followed by infiltration of LR White (EMS) alone for 4 h and a final change of LR White followed by overnight incubation at 4 °C. Embedding in LR white was done in gelatin capsules at 50 °C for 24 h in an oven. Ultrathin sections were cut and collected on carbon-coated Formvar films mounted on single slot copper grids (2 × 1 mm; EMS).

#### 4.8.2. Immunofluorescence on Ultrathin Sections

The above described ultrathin sections of transduced HepG2 cells embedded in LR White were incubated for 20 min with 50 mM Glycine before blocking for 30 min with blocking buffer (0.5% BSA/0.1% gelatin (Cold Water Fish Skin, EMS, Hatfield, PA, USA)) followed by 5 min incubation in 0.1% acetylated BSA (BSA-c) (Aurion, Wageningen, The Netherlands), pH 7.5 at room temperature (RT). Incubation was done overnight with the polyclonal goat anti-RV serum (obtained from Catherine Eichwald, University of Zurich, Zurich, Switzerland) diluted 1:10 in 0.1% BSA-c at 4 °C. After washing five times with BSA-c, the sections were incubated with the secondary anti-goat antibody conjugated with Alexa Fluor 488 (Molecular Probes), diluted 1:500 in 0.1% BSA-c for 1 h at RT. After washing 5 times with 0.1% BSA-c and once with H_2_O, the sections were stained with DAPI (0.1 mg/mL) for 15 min followed by 2 washes with PBS. After the staining, the sections were sandwiched between a microscope slide and a coverslip in PBS and sealed with nail polish. With a fluorescence microscope (Axio Observer inverted microscope), the whole grid was imaged in bright field and fluorescence and several images were taken with a 100× oil (NA = 1.25) objective. These images served as maps and were then used to find the same areas in the scanning electron microscope (SEM). 

#### 4.8.3. Scanning Electron Microscopy 

The grids are recovered from the fluorescent microscope settings and further processed for electron microscopy. The ultrathin sections were stained with uranyl acetate and lead citrate. Finally, the grids are loaded into the scanning transmission electron microscope (STEM) holder of the SEM (Helios NanoLab 650, FEI Company, Eindhoven, The Netherlands). The images taken with the fluorescence microscope were opened on the SEM computer using the Maps software (FEI Company, Eindhoven, The Netherlands), a specially designed software to correlate light and electron microscopy which can read any type of images obtained by any type of light microscope. An electron micrograph was recorded using the Everhart–Thorney secondary electron detector and the alignment between the light and the electron micrograph was done using the Maps software using two auspicious points. A grid of image tiles, tileset, was drawn over the area of interest, indicated by the fluorescence image. The tile size was determined by the imaging conditions and had an overlap of 10%. Imaging conditions were: 30 keV, 1.6 nA, 5 mm working distance, 6144 × 4096 pixels per frame, 5 µm horizontal field of view (about 8 Å pixel size), 1 µs dwell time using the STEM III detector (FEI Company) in the high-angle annular-dark field mode. We used the three-point focus regime: at three points close to the area of interest the section was focused at higher magnification, then brightness and contrast were adjusted at the magnification and imaging conditions used for recording the images. Finally, the tiles were stitched by the software and exported as tiff files. To circumvent any disturbing effects (e.g., bleaching), occurring in the overlapping zones after stitching, the whole area of interest was pre-irradiated with a high current (26 nA), large frame size (6144 × 4096 pixels) and short dwell time (50 ns) for about 30 min.

### 4.9. Immunization of Mice and Sample Collection

All animal procedures were conducted in accordance with the regulations of the Swiss Federal Committee on Animal Experimentation and with the Veterinary Office of the Canton Zurich (approval number: 70/2014). Six-to-eight-week-old naïve female BALB/c mice were i.m. inoculated at days 0 and for the first set of experiments at day 19 post-prime and for the second set of experiments 20 days post-prime with either the indicated HSV-1 amplicon vectors (10^6^ TU per animal per dose) or HBSS. Five days after the prime immunization, mice were mated by adding one male to two females per cage. Since multiple blood sampling was done regularly over a period of about two weeks, only small blood samples (5–10 µL) were taken at each sampling via tail vein punctuation and collected using 20-µL microcaps capillary tubes (Sigma–Aldrich). Mice were milked as described previously [50]. Briefly, dams were separated from their offspring for about 2 h and then anesthetized. Oxytocin (100 µL of 10 IU/mL) was injected sub-cutaneous (s.c.) and milk flow was stimulated manually and collected using microcaps capillary tubes.

### 4.10. Detection of Antibody Responses by Enzyme-Linked Immunosorbent Assay

Serum and milk IgG was analyzed by an indirect ELISA as described previously [50]. Briefly, ELISA plates were coated with sucrose concentrated wt RV (strain Wa, diluted 1:100 in carbonate buffer), washed with PBS-Tween (phosphate buffered saline with 0.05% Tween 20) and incubated with the samples. Serum samples were diluted to 1:5000 and milk samples to 1:500 before application. After washing with PBS-Tween, wells were incubated with the HRP-conjugated goat anti-mouse IgG antibody (Pierce Biotechnologies, Rockford, IL, diluted 1:8000) and analyzed using peroxidase substrate (TMB substrate solution, Thermo Scientific, Waltham, MA). Reaction was stopped with 2 M H_2_SO_4_ and the optical density was measured with an ELISA microplate reader. In order to compare values from different ELISA plates, a serum pool of a RV hyper immunized mice from previous studies [17] was used to normalize the values taking the hyper immune sera as 100%.

For the evaluation of IgG isotypes (IgG1, IgG2a and IgG2b) standardized biotinylated isotype-specific monoclonal antibodies (IgG1: RMG1-1, 0.1 μg/mL, IgG2a: RMG2a-62, 0.2 μg/mL, IgG2b: RMG2b-1, 1.6 μg/mL, all from BioLegend, San Diego, CA, USA) and for detection HRP-conjugated streptavidin (1:2000, BioLegend, San Diego, CA, USA) were used. For the standardization IgG1; IgG2a; IgG2b monoclonal immunoglobulins were first coated in identical concentrations directly to ELISA plates as antigen, followed by various dilutions of the detection system and appropriate concentrations of the antibodies (given above) selected. Serum samples of dams and their pups (diluted 1:1000) were reanalyzed with the antigen coated as above but the IgG isotypes were detected with the appropriate standardized detection system.

### 4.11. Virus Challenge of Newborns

Rotavirus challenge of 2- to 4-days-old suckling mice from all groups was performed by oral gavage of 30 µL of virus-containing solution (strain EDIM, 10× DD_50_) using a feeding needle. Pups were monitored twice a day for RV symptoms (diarrhea and dehydration) as well as for overall physical appearance. They were considered sick when yellow and liquid stools appeared upon gentle abdominal palpation.

### 4.12. Reverse Transcription Quantitative Real-Time Polymerase Chain Reaction 

Quantitative real-time polymerase chain reaction (RT-qPCR) was performed directly from serum samples. The serum samples were 1:5 diluted in 1× PBS and heated for 3 min at 97 °C, cooled immediately on wet ice for 5 min and used directly for the RT-qPCR reaction. The Path-ID Multiplex One-Step RT-PCR Kit (Applied Biosystems, Thermo Fisher Scientific) was used for the amplification. As primer probe, we used the VetMax Swine Enteric Panel Reagent (Applied Biosystems, Thermo Fisher Scientific), a primer probe mix for detection of porcine rotavirus A—besides a primer probe for porcine rotavirus A, this kit contains also primer probes for transmissible gastroenteritis coronavirus (TGEV) and porcine epidemic diarrhea virus (PEDV)—. This primer probe mix was selected because it was the most sensitive kit. The reaction was run in a thermal cycler (CFX96 C1000 Touch, BioRad, Hercules, CA, USA) using the following settings: reverse transcription for 10 min at 48 °C followed by an inactivation/initial denaturation step of 10 min at 95 °C. Amplification was performed as 40 cycles of 15 s at 95 °C and 45 s at 60 °C. Reactions were done in duplicates. For calculation of virus dose, a standard curve was drawn using serial dilution of EDIM virus diluted in negative mouse serum and treated as described above in triplicates. Based on the resulting formula of the determined standard curve (ln(DD50) = −0.6419 × Cq + 16.565), DD_50_ was calculated for each sample. Considering the applied volume of the analyzed samples, DD_50_/mL was determined (DD_50_/0.0016 mL).

### 4.13. Statistical Analysis

Enzyme-linked immunosorbent assay measurements were analyzed as follows: The mean of each sample triplicate was calculated and the mean value of the negative control from the same plate was subtracted. To make values between the plates comparable, always the same positive control was applied on each plate. The mean of each sample triplicate was then set in relation to the mean of the positive control (100%) from the individual plate. A two-way ANOVA with weighted means was performed to calculate the *p*-values. For samples of Figure 3D, Tukey ad hoc testing was done to determine the *p*-values between all groups. Pre-processing of data (normalization, calculation of means and standard deviations) was performed with Microsoft Excel 2011 (Version 14.1.0). Two-way ANOVA with weighted means and Tukey ad hoc testing were performed using the open source program RStudio (Version 0.99.903—2009–2016 RStudio, Inc., Boston, MA, USA). Graphs were generated with RStudio.

## 5. Conclusions

The main aim of this study was to evaluate a safe RV-specific vaccine based on the non-replicating HSV-1 amplicon vector system to induce protective systemic antibodies in a first attempt. For this, the RV proteins were used in a structural vaccinology approach. The transduction with the RV protein encoding HSV-1 amplicon vector sWa[VP2/6/7] triggered the synthesis of all three encoded RV proteins, VP2, VP6 and VP7, as well as the intracellular assembly of RVLPs in cell culture. The distribution of the viral proteins within the cell resembled the localization of proteins in wild type RV infections. When administered intramuscularly, the RV proteins encoding HSV-1 amplicon vectors induced a strong RV specific antibody response in sera as well as in milk of vaccinated dams. In addition, the antibody response was passed through both the placental and/or lactogenic route to unvaccinated offspring swapped to vaccinated dams. As expected, lactogenic transfer of RV specific IgG did not protect the suckling mice from developing RV symptoms upon challenge. Indications of control of systemic spread were noted but will require more targeted experiments. Although full protection against an oral RV challenge was not achieved and would require both oral and systemic vaccination, the helpervirus-free herpesvirus amplicon vectors used in this study may be rapidly adapted to confer immunity against a whole array of different RVs.

## Figures and Tables

**Figure A1:**
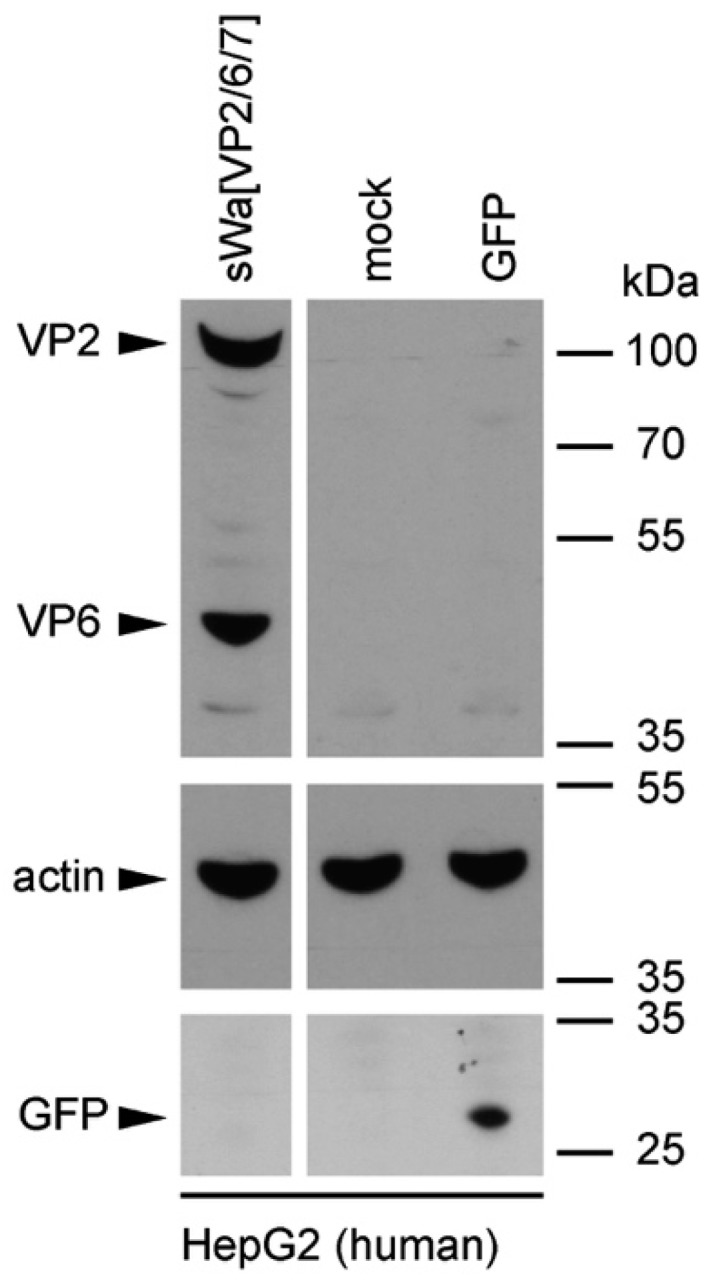
Analysis of guinea pig anti-RV serum specificity. Western blot analysis of whole HepG2 cell lysates from sWa[VP2/6/7] or GFP amplicon vector transduced (MOI 2) or non-transduced mock cells. Proteins were analyzed using the guinea pig polyclonal anti-RV antibody (upper panel); anti-actin antibody (middle panel); or the anti-GFP antibody (lower panel). Expected migration lengths of the RV proteins VP2 and VP6, actin and GFP are indicated on the left with arrows and the positions of the molecular weight markers are shown on the right in kDa.

## Appendix A

**Table A1:** This table shows all data used to generate Figure 3B as well as the number of animals included in this study and the *p*-values.

Group	dpb	rIgG (%)	SD	No. Animals	*p*-Value ^1^
sWa[VP2/6/7]	−19	1.449	1.213	6	0.3025
GFP	−19	0.693	0.227	4	
sWa[VP2/6/7]	0	3.570	0.730	6	1.40 × 10^−5^
GFP	0	0.846	0.264	6	
sWa[VP2/6/7]	10	54.852	13.770	7	0.002028
GFP	10	2.147	0.311	2	
sWa[VP2/6/7]	14	37.893	11.319	6	0.00011
GFP	14	1.383	0.775	5	
sWa[VP2/6/7]	20	23.777	8.309	2	0.03876
GFP	20	1.916	0.338	3	

^1^ *p*-values were calculated applying a two-way ANOVA. Measurements from sWa[VP2/6/7]-immunized versus GFP-immunized animals are compared. dpb: Days post-booster immunization; rIgG: Relative RV specific IgG; SD: Standard deviation.

**Table A2:** This table displays all data used to generate Figure 3C as well as the number of animals included in this study and the *p*-values.

Group	dpd	rIgG	SD	No. Animals	*p*-Value ^1^
GFP	0	1.774	1.044	3	0.008351
sWa[VP2/6/7]	0	42.567	14.131	4	
GFP	3	2.289	0.612	3	1.58 × 10^−5^
sWa[VP2/6/7]	3	57.135	6.547	5	
GFP	6	1.800	0.210	3	0.001049
sWa[VP2/6/7]	6	50.328	12.327	5	

^1^ *p*-values were calculated with a two-way ANOVA comparing the measurements from sWa[VP2/6/7]-immunized versus GFP-immunized animals.

**Table A3:** This table displays all data used to generate Figure 3D as well as the number of animals included in this study and the *p*-values.

Group	dpd	rIgG	SD	No. Animals	*p*-Value ^1^
GFP	0	1.236	0.509	3	0.001761
sWa[VP2/6/7]	0	46.640	10.952	4	
GFP	3	2.253	0.659	3	0.001214
sWa[VP2/6/7]	3	57.263	14.364	5	
GFP	6	1.966	0.565	3	0.004806
sWa[VP2/6/7]	6	51.828	17.176	5	

^1^ *p*-values were calculated with a two-way ANOVA comparing the measurements from sWa[VP2/6/7]-immunized versus GFP-immunized animals.

**Table A4:** Optical density values of IgG isotype ELISA from serum samples (diluted 1:1000) of sWaRV[VP2/6/7] or GFP control immunized animals.

Group	Animal	IgG1	IgG2a	IgG2b
sWaRV[VP2/6/7]	Dam 1	0.072	0.274	0.077
sWaRV[VP2/6/7]	Pups Dam 1	0.104	0.389	0.042
sWaRV[VP2/6/7]	Dam 2	0.086	0.122	0.018
sWaRV[VP2/6/7]	Pups Dam 2	0.201	0.367	0.033
sWaRV[VP2/6/7]	Dam 3	0.086	0.337	0.014
sWaRV[VP2/6/7]	Pups Dam 3	0.163	0.612	0.013
sWaRV[VP2/6/7]	Dam 4	0.035	0.114	0.011
sWaRV[VP2/6/7]	Pups Dam 4	0.075	0.370	0.010
GFP	Dam 5	0.016	0.026	0.009
GFP	Pups Dam 5	0.017	0.020	0.014
GFP	Dam 6	0.024	0.018	0.013
GFP	Pups Dam 6	0.033	0.025	0.012
GFP	Dam 7	0.0096	0.014	0.010
GFP	Dam 8	0.037	0.029	0.012
GFP	Pups Dam 7/8	0.030	0.045	0.010

**Table A5:** Ratio of IgG isotype values from sWaRV[VP2/6/7] immunized animals from Table A10.

Group	Animal	IgG1/IgG2a	IgG1/IgG2b
sWaRV[VP2/6/7]	Dam 1	0.263	0.934
sWaRV[VP2/6/7]	Pups Dam 1	0.267	2.491
sWaRV[VP2/6/7]	Dam 2	0.707	4.803
sWaRV[VP2/6/7]	Pups Dam 2	0.549	6.110
sWaRV[VP2/6/7]	Dam 3	0.256	5.977
sWaRV[VP2/6/7]	Pups Dam 3	0.266	12.253
sWaRV[VP2/6/7]	Dam 4	0.305	3.229
sWaRV[VP2/6/7]	Pups Dam 4	0.203	7.426

**Table A6:** This table shows the relative IgG values of serum samples of the dams from experiment 2. These dams raised the pups further analyzed in Figure 3E.

Group	dpb	rIgG (%)	SD	No. Animals
GFP	−20	1.327	0.163	6
GFP	0	1.899	0.419	6
GFP	8	4.144	0.684	6
GFP	11	2.786	0.552	2
GFP	12	3.361	0.685	4
HBSS	−20	0.741	0.682	10
HBSS	0	0.448	0.409	8
HBSS	5	0.705	0.518	2
HBSS	6	0.610	0.573	3
HBSS	9	0.242	0.149	3
HBSS	11	2.224	1.822	2
HBSS	12	1.751	1.036	3
HBSS	15	1.171	1.306	3
sWa[VP2/6/7]	−20	0.816	0.639	6
sWa[VP2/6/7]	0	4.452	2.633	6
sWa[VP2/6/7]	7	32.853	0.000	1
sWa[VP2/6/7]	8	37.313	5.621	3
sWa[VP2/6/7]	9	40.592	0.534	2
sWa[VP2/6/7]	11	46.500	0.000	1
sWa[VP2/6/7]	14	29.957	2.138	3
sWa[VP2/6/7]	15	33.809	1.109	2

**Table A7:** This table shows the relative IgG values of milk samples of the dams from experiment 2. These dams raised the pups further analyzed in Figure 3E.

Group	dpb	rIgG (%)	SD	No. Animals
GFP	0	61.600	1.661	2
GFP	2	52.830	10.911	2
HBSS	0	0.575	0.141	4
HBSS	1	0.279	0.051	4
HBSS	2	0.263	0.120	2
HBSS	6	0.431	0.128	2
HBSS	7	0.378	0.129	3
HBSS	8	0.271	0.000	1
sWaRV2/6/7	1	57.312	8.009	5
sWaRV2/6/7	2	40.092	0.336	2
sWaRV2/6/7	6	42.919	0.000	1
sWaRV2/6/7	7	39.086	6.285	4
sWaRV2/6/7	8	31.470	0.000	1
GFP	0	61.600	1.661	2
GFP	2	52.830	10.911	2

**Table A8:** This table shows all data used to generate Figure 3E as well as the number of animals included in the study. *p*-values are shown in Table 7A.

Group	rIgG	SD	No. Animals
A	21.103	4.023	4
B	25.386	6.109	6
C	4.421	1.032	8
D	0.465	0.426	13
E	2.461	0.441	31

**Table A9:** This table shows the *p*-values between the indicated groups from the data used for Figure 3E and the data displayed in Table A6.

Compared Groups	*p*-Value ^1^
B-A	0.5688202
C-A	0.0006268
D-A	0.0000157
E-A	0.0000537
C-B	0.0000129
D-B	0.0000002
E-B	0.0000006
D-C	0.6376254
E-C	0.9552024
E-D	0.9224124

^1^ *p*-values were calculated using the Tukey ad hoc test.

**Table A10:** This table shows the quantification cycle (Cq) values of pooled serum samples of pups analyzed by RV-specific RT-qPCR.

Group	Cq 1	Cq 2	Cq 3	Cq 4	Mean Cq	DD_50_/mL
A: Pups born and raised by sWa[VP2/6/7]-immunized dams	-	-	38.235	36.055	37.145	4.314
B: Pups born from HBSS-injected dams but raised by sWa[VP2/6/7]-immunized dams	29.28	34.81	33.39	30.41	31.973	119.365
C: Pups born from sWa[VP2/6/7]-immunized dams and raised by HBSS-injected control dams	-	33.77	31.11	28.445	31.108	207.867

DD_50_: Diarrhea dose 50.

**Table A11:** This table summarizes the data and variables obtained during the challenge experiment shown in Figure 3E.

Group	Litter Size	Pup Age at Challenge (Days)	Time until Diarrhea (Days)	Pup Age at Sampling (Days)	Mean Cq from Triplicates	DD_50_	DD_50_/mL
A	3	4	2.5	6.5	38.235	0.003	2.143
A	3	3	2	5	36.055	0.014	8.685
B	4	4	2	6	29.280	1.075	672.166
B	4	4	2.5	6.5	34.810	0.031	19.313
B	3	5	2	7	33.390	0.077	48.053
B	4	4	2	6	30.410	0.521	325.433
C	4	4	2.5	6.5	33.770	0.060	37.652
C	6	4	2	6	31.110	0.332	207.645
C	5	5	2	7	28.445	1.838	1148.821
D	6	4	1.5	5.5	33.880	0.056	35.085
D	7	4	1.5	5.5	31.770	0.217	135.935
D	3	3	2.5	5.5	36.200	0.013	7.913
D	4	3	2	5	35.590	0.019	11.706
D	6	4	1.5	5.5	30.950	0.368	230.104
E	8	2	2	4	34.430	0.039	24.649
E	8	2	2.5	4.5	35.405	0.021	13.182
E	4	3	2	5	34.885	0.029	18.406
E	5	3	2.5	5.5	32.680	0.121	75.796
E	6	4	2.5	6.5	32.520	0.134	83.994

Group A: Offspring born and raised by sWa[VP2/6/7]-immunized dams; Group B: Offspring born from HBSS-injected dams but raised by sWa[VP2/6/7]-immunized dams; Group C: Offspring born from sWa[VP2/6/7]-immunized dams and raised by HBSS-injected control dams; Group D: Offspring born and raised by HBSS-injected control animals; Group E: Offspring born and raised by GFP vector-immunized dams.
